# Healthcare Models and Quality Indicators in the Management of Patients with Heart Failure in Spain: Results from the CARABELA-HF Initiative

**DOI:** 10.3390/jcm14103378

**Published:** 2025-05-12

**Authors:** Inmaculada Mediavilla, Manuel Anguita, Álvaro González Franco, Manuel Leal, CARABELA-HF Scientific Committee, José Francisco Soto

**Affiliations:** 1 Gerencia Asistencial de Atención Primaria, Servicio Madrileño de Salud, 28046 Madrid, Spain; 2 UGC de Cardiología, Hospital Universitario Reina Sofía, IBIMIC, Universidad de Córdoba, 14004 Córdoba, Spain; 3 CIBER Cardiovascular, Instituto San Carlos III, 28029 Madrid, Spain; 4 Hospital Universitario Central de Asturias, 33011 Oviedo, Spain; 5 Medical Department, AstraZeneca Farmacéutica Spain, 28050 Madrid, Spain; 6 Gerencia Fundación Hospital Instituto San José, 28054 Madrid, Spain

**Keywords:** heart failure, healthcare models, indicators, quality of care, management

## Abstract

**Background/Objectives:** Heart failure (HF) poses a significant global health burden. In Spain, its prevalence rises annually, contributing significantly to cardiovascular-related hospitalizations and deaths. Through a broad and integrative perspective, the CARABELA-HF initiative seeks to improve the organization and delivery of HF care in Spain, addressing the key challenges identified across the care continuum. **Methods:** CARABELA-HF involved four phases: characterization of HF care models, validation of improvement areas, potential solutions and healthcare quality indicators, refinement of results from a regional perspective, and local dissemination and implementation. Ten pilot centers participated, and nine variables were identified to characterize operating HF care models. **Results:** Four HF care models were identified based on the degree of coordination between departments and resource availability. Structure, quality of care, and transformation indicators were used to evaluate these models, revealing improvement areas. Overall, this process identified solutions for generating a comprehensive and integrated HF care model, highlighting enhanced coordination, digital transformation, enhanced nursing roles, professional training and patients’ education, accredited HF care models, resource accessibility, and data-based evaluation. **Conclusions:** CARABELA-HF provides insights into current HF care models in Spain and identifies healthcare quality indicators for future improvement efforts. It strives to enhance patient outcomes, raise healthcare standards, and improve overall system efficiency through the promotion of a comprehensive and integrated HF care pathway.

## 1. Introduction

There is a pressing need for strategies that optimize the management of heart failure (HF), a common condition that is the leading cause of premature death worldwide [1]. The disease burden is very high, and its prevalence is progressively increasing for reasons that include, among others, the aging of the population and the greater coexistence of risk factors [2]. In Spain, the estimated prevalence of HF is 2% according to recently published studies, but this rate is increasing every year [3]. This disease is also a major contributor to cardiovascular-related hospitalizations and deaths [4]. It is the principal cause of hospital admissions in people older than 65 years [5] and 50% of patients die in the first five years after diagnosis [4]. In terms of economic impact, HF accounts for 2% of Spanish health expenditure [6], but this figure could rise as prevalence increases.

The CARABELA-HF initiative addresses the improvement areas in the management of HF in the healthcare system in Spain. CARABELA initiatives are led by scientific societies and AstraZeneca to analyze and tackle the current situation and needs in chronic conditions with the aim of driving a holistic transformation toward systemic and practical improvement. CARABELA-HF is therefore a transversal initiative that seeks to reduce existing management inequalities and inefficiencies, improve the quality of care, and optimize the routine management of HF, taking into account the various realities and care models present in Spain [7]. A recent publication introduced CARABELA-HF as a joint collaboration between scientific societies and AstraZeneca and described the care of patients with HF in Spain as a circular multidisciplinary process where primary care, internal medicine and cardiology go hand-in-hand, in a patient-centered approach [8].

We report here the results obtained during the CARABELA-HF initiative, which aimed to analyze current HF management models in Spain in order to develop healthcare quality indicators for detecting improvement areas and defining lines of action.

## 2. Methods

### CARABELA-HF Design and Participants

This initiative has been developed and coordinated throughout Spain by the main scientific societies involved in the management of HF in Spain: Spanish Society of Cardiology (SEC), Spanish Society of Internal Medicine (SEMI), Spanish Society of Quality of Care (SECA), and Spanish Society of Health Managers (SEDISA). A total of 10 pilot centers participated in the characterization phase. The overall structure and methodological foundation of the CARABELA initiative has been detailed previously [7]. In the case of CARABELA-HF, the project was organized into four phases:

Phase 1: Characterization and evaluation of HF healthcare models in Spanish hospitals.

The characterization phase was grounded in the concept of HF care as a cyclical and multidisciplinary process [8]. The current models of HF clinical management were analyzed in the 10 pilot centers and presented in sessions in which healthcare professionals from each center participated. Data collection at each center was conducted via structured working sessions involving multidisciplinary clinical teams. Information was gathered regarding coordination practices, resource availability, and professional roles in HF management. In these meetings, a total of 9 variables were selected by the Scientific Committee based on clinical relevance and feasibility of collection. These helped to categorize the different models of patient care and define the type and degree of coordination between the medical specialties involved in HF care, the roles of implicated professionals, and the availability of certain resources: coordination between cardiology and internal medicine; availability of skilled nursing staff; availability of nurse consultations; responsibility for drug titration; availability of case managers; use of HF protocols and circuits; role of other specialties; existence of multidisciplinary committees; and presence of a day hospital. The identification and classification of the four HF care models was based on the comparative analysis of these variables across centers. Data on these nine variables were collected through structured working sessions held with multidisciplinary teams from each pilot center, including cardiologists, internists, and nursing staff.

A set of healthcare quality indicators was created to assess each identified model and to establish a reference framework for future monitoring, through iterative consensus among the CARABELA-HF scientific committee, using structured expert meetings, as well as national and regional workshops. These indicators were subsequently piloted in the participating pilot centers and were divided by type into three main clusters: structure, quality of care, and transformation.

Phase 2 (validation), Phase 3 (cocreation), and Phase 4 (dissemination and implementation).

During Phase 2, the identified models and the interpretation of the data collected during Phase 1, were reviewed and used to identify improvement areas and potential solutions that were subsequently validated by members of the Spanish HF health ecosystem during the National CARABELA-HF Conference through a structured consensus process.

Finally, all the results obtained were synthesized and the regional refinement of solutions to address improvement areas in the HF care process was drawn up during the co-creation Phase 3. In Phase 4, the analyses and potential solutions were disseminated to as many Spanish healthcare centers as possible. These results were included in a digital questionnaire in the form of a playbook.

All collected information was descriptive and qualitative, aimed at capturing the organizational features of each center rather than obtaining quantitative measurements or collecting patient-level clinical outcome data such as mortality or readmission rates. No formal measurement scales or standardized instruments were used, nor were formal statistical analyses applied.

## 3. Results

### 3.1. Coordination Models for the Management of HF in Spanish Hospitals

**Figure 1:**
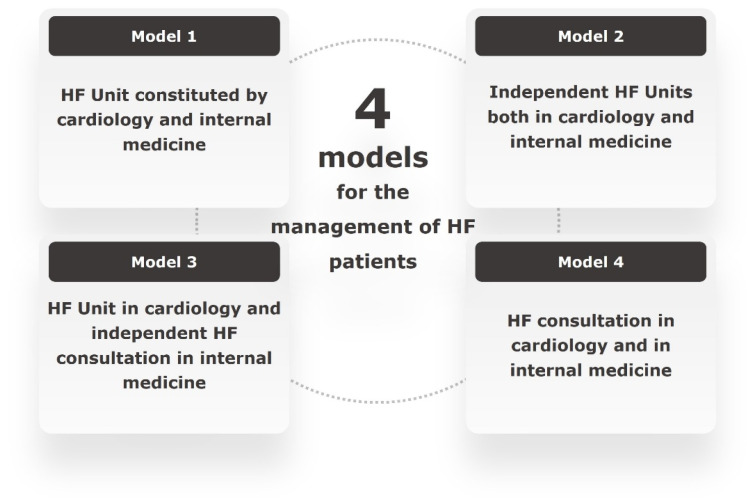
Identified models for the care of HF patients.

During the pilot phase, a total of four care models of the HF patient were identified which mainly differed on the degree of coordination and communication between the departments involved in patient management. The availability of resources for HF management among the models were notably different. An overview of the four models is shown in Figure 1 and in the Supplementary Materials.

**Figure 2:**
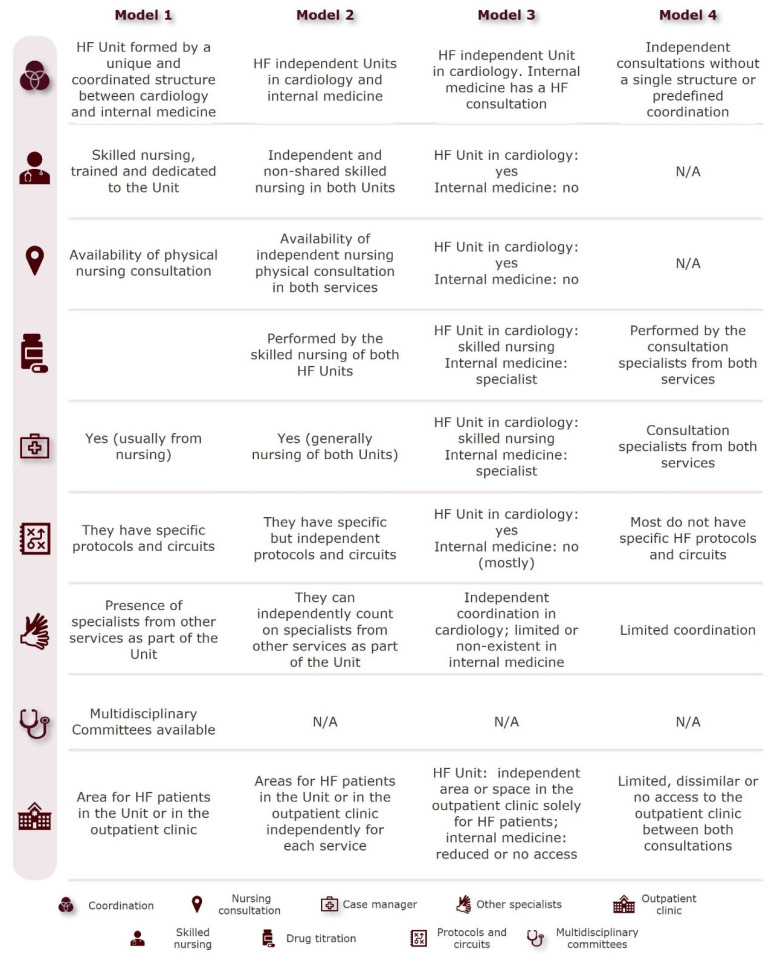
Comparison of the characterized models for the management of HF.

Model 1 consists of a single HF unit integrating cardiology and internal medicine, with equal resource access, a skilled and trained nursing team, a dedicated consultation room, a designated person responsible for drug titrations, and a case manager overseeing patient transitions in and out of the hospital. The unit applies specific protocols and circuits and has multidisciplinary committees and specialists from other departments. Model 2 consists of two independent HF units within cardiology and internal medicine, with unequal access to resources, a lower level of coordination than Model 1 and no predetermined multidisciplinary committees discussing patient management. Model 3 consists of an HF unit in the cardiology department, while internal medicine relies on external HF consultations, and has less access to resources. Only the HF unit within cardiology has a specific protocol and circuit for HF care and an independent area within the day hospital. Model 4 lacks structured HF units relying on independent consultations for HF patient management without interdepartmental coordination or access to resources, and specialists from each department are responsible for tasks such as drug titration and case management. This model lacks skilled nursing staff, specific protocols and circuits are rare, and coordination with other specialties is limited. Access to the day hospital is scant and varies between departments. Model characteristics are compared in Figure 2.

### 3.2. Indicators to Evaluate Care Models

HF units must be assessed from different perspectives. Care models must therefore be analyzed on the basis of three indicator types (structure, quality of care, and transformation) that have been previously described in detail [7]. Conclusions can be drawn about the model by gathering and evaluating predetermined indicators of each type.

The structure indicator category focuses on the organization of HF management in the center and covers four areas: hospital environment; resources in the HF unit; origin and characteristics of patients with HF; and care process. Within this category, the analysis defined indicators such as the number of HF patients in the unit, the number of different healthcare professionals, indicators related to the proportion of time invested on consultation, diagnostic tests, and patient education, the percentage of referrals from primary care or other specialties and waiting lists. All structure indicators are listed in Table 1.

Healthcare quality indicators were categorized based on the phases of the care process: suspicion (i.e., >70% of patients with HF suspicion are referred with a digitized electrocardiogram), diagnosis (i.e., virtual consultation between primary care and specialists), treatment (i.e., treatment adherence programs), and follow-up (i.e., skilled HF nursing staff) (Table 2).

Likewise, the scientific committee defined three types of transformation indicators to evaluate the care model evolution: transformation of the structure; transformation of the process; and health outcomes (Table 3).

This subdivision included the presence of a cardiac intensive care unit, a person in the hospital who is responsible for the centralization of patient transitions, the percentage of confirmed diagnosed patients referred from primary care, and the average length of stay in the cardiology or internal medicine ward of patients admitted with a HF diagnosis, among others.

### 3.3. The Future of HF: Improvement Areas for the Evolution of Care Models, Barriers, and Potential Solutions

The in-depth analysis of the identified care models led to the recognition of 13 areas of improvement associated with the distinct phases of the HF care process (suspicion, diagnosis, treatment, and follow-up) that were validated during the National CARABELA-HF Conference. These improvement areas would be addressed in the future for the design of an integrated care model. The initial management of patients with HF suspicion was identified as an improvement area at the clinical suspicion level, along with the development of an integrated model for HF management at diagnosis level, the standardization of patient management irrespective of the responsible department at treatment level and the implementation of a coordinated management model between different care levels with standardized follow-up of HF patients at follow-up level.

Finally, after characterizing the different care models in place throughout Spain, identifying the areas of improvement with the greatest potential impact both nationally and regionally, and gathering the perspectives of healthcare professionals through meetings conducted as part of the CARABELA-HF initiative, we identified key solutions that should be pursued to develop a comprehensive and integrated HF care model: (1) coordination and protocols, (2) a virtual care model and digital transformation, (3) roles and functions of the nursing team, (4) professional training and patient education on HF, (5) access to resources, (6) accredited care models, and (7) registries and data evaluation.

## 4. Discussion

The need for collaborative HF management is well known. In fact, the SEC and the SEMI have been promoting the establishment of specialized units in Spanish hospitals for years [9], and the European Society of Cardiology (ESC) recommends that patients with HF should be treated in multidisciplinary units to reduce hospitalizations and mortality [10]. This is partly because in the management of HF, the role of the internist is crucial, as many patients present with multiple comorbidities that are treated by internal medicine. Furthermore, HF units should be run systematically, as proposed in a scientific consensus published by the SEC in 2016. This document defined both quality standards and methods for the accreditation of HF units [11]. Further down the line, we must now assess the implementation of these quality-of-care programs and produce an overview of how HF is managed in Spanish centers nowadays. For this reason, the CARABELA-HF initiative has focused on defining the shared model between cardiology and internal medicine and on identifying the use of resources and the management characteristics. Only in this way can limitations be detected, and solutions designed.

This detailed characterization process implemented in 10 centers nationwide has identified that while the range of available resources determines the type and quality of care provided to patients, the main differentiating factor in the type of care received is the coordination and communication between cardiology and internal medicine. Inadequate coordination or delayed referral, more characteristic of Models 3 and 4, can hinder timely optimization of pharmacological therapy, which has been linked to increased morbidity and higher readmission rates [2,10]. Equitable access to the center’s resources is clearly key in generating a model of coordination and optimal communication between units in the care of patients with HF, while the collaboration and integration of other specialties into the unit offers a 360° vision. The effectiveness of implementing multidisciplinary HF management programs with an equal allocation of assets between units has been proven in other countries [12]. Another conclusion was that periodically convening multidisciplinary clinical case committees is useful for improving the care of patients with HF. Considering the impact of HF on the Spanish population [5,13], the early optimization and use of disease-modifying drugs is deficient. Prompt horizontal introduction of these therapies, rather than a staggered approach, would avoid a delay in the administration of highly effective therapies. All centers recognize the need to generate rapid care circuits for HF patients, including medical record flagging to streamline their journey throughout the care process. In terms of resources, the centers generally do not have a professional with predefined roles and functions who is responsible for the management of internal/external patient transitions throughout the care process. Furthermore, the availability of specialized, fully dedicated nurses with their own consulting room appears to be key to improving patient care both during treatment and at follow-up. This type of organizational structure, characterized by strong coordination between specialties, equitable access to resources, and the involvement of HF-specialized nurses, has been associated with better clinical outcomes. For instance, accredited HF units in Spain, which share many features with Model 1, have demonstrated improved adherence to guideline-directed medical therapy and more systematic follow-up strategies, contributing to higher quality of care [9].

Although CARABELA-HF was conducted within the specific organizational framework of the Spanish healthcare system, several principles identified may be broadly applicable across different countries, despite differences in healthcare structures and financing models. These include the critical role of structured multidisciplinary collaboration, the early initiation and optimization of disease-modifying therapies, the importance of consistent follow-up strategies, and the equitable allocation of healthcare resources. Moreover, disease management programs and multidisciplinary care approaches have shown to reduce hospital readmissions and improve survival and patient quality of life [2,14]. Prioritizing models with stronger integration and resource access, and adapting these principles to local contexts, may contribute to improved HF care delivery and outcomes worldwide. This approach aligns with the ESC guidelines, which recommend early optimization of pharmacological treatment, patient education, and care integration as essential strategies to improve long-term outcomes in HF [10].

The main limitation of this initiative is its lack of quantitative methodology, but notwithstanding, the use of the healthcare quality indicators described in this document as an instrument for evaluating care models was fundamental for analyzing the efficacy of our processes in terms of organization, structure, resources, and patient empowerment. The previous literature has reported that a system of indicators to measure intervention outcomes and to ensure continuous improvement in the quality of care is essential for the success of disease management programs [14]. To that end, indicators must be synthesized and endorsed at a national level, as isolated databases are insufficient. The CARABELA-HF initiative has established a system of indicators that covers all aspects of the care process and can be used systematically at a national level.

CARABELA-HF offers the added value of a public and private multidisciplinary collaboration involving numerous national experts and several scientific societies focused on the quality management of HF. We have included not only entities with a clinical perspective, but also institutions from the healthcare management field, as the latter are indispensable for the organization of healthcare provision. Strengthening collaboration with primary care is assuredly the great improvement area for the near future [9], and ongoing initiative within the CARABELA “fleet” are focused on this approach.

## 5. Conclusions

This joint CARABELA initiative between scientific societies and AstraZeneca identifies the current models of care of HF in Spanish hospitals and defines the healthcare quality indicators that must be used for their evaluation. It also creates a framework for promoting improvement and enhancing coordination between different care levels and specialties, approaching each patient in an individualized manner. This, in turn, will result in better outcomes, better healthcare standards, and the improved efficiency of the whole healthcare system.

## Figures and Tables

**Table 1:** Structure indicators identified to evaluate care models in the CARABELA-HF initiative.

Area	Indicator
**Hospital environment**	Existence of emergency protocols for patients with exacerbations (Yes/No) Number of patients in the HF unit Number of available beds Hospital reference population University hospital (Yes/No) Number of clinical trials in HF
**Resources of the HF unit**	**Number of healthcare professionals (HP)**
	HP from cardiology HP from cardiac surgery HP from internal medicine HP from primary care Resident HP HP from other specialties HP from nursing
	**Proportion of the working day spent by medical staff on the following activities (in %):**
	Appointment management HF consultations Diagnostic tests Day hospital Management of acute patients with advanced HF Patient education and follow-up Coordination between specialties Inpatient care
	**Availability of facilities**
	Number of offices Nursing rooms Diagnostic rooms Capacity of the day hospital Capacity of the critical care unit
	**Daily and weekly planning of the following consultations:**
	General cardiology Cardiology from the HF unit
**Origin and characteristics of patients with HF**	**Impact of HF on the hospital**
	Number of admitted patients Total number of patients with HF % of patients with >1 admission due to HF in the 12 months after discharge Number of heart transplants Annual in-hospital mortality Readmissions rate at 30 days after discharge Number of short-length vascular accidents Number of long-length vascular accidents
	**Percentage of patients referred from:**
	Emergency room Primary care Cardiology Outpatient visits Other specialties
**Care process**	**Number of annual diagnostic tests and waiting list for the following tests:**
	Analysis of natriuretic peptides Cardiac catheterization Nuclear magnetic resonance Coronary CT scan Transthoracic ultrasound
	**Parameters to be measured with respect to care activity:**
	Number of weekly consultations in the HF unit Waiting lists in the first and subsequent consultations Time spent on first and subsequent consultations

**Table 2:** Healthcare quality indicators defined in the CARABELA-HF initiative for the distinct phases of the care process.

**Suspicion**
>70% of patients with suspected HF referred with digitized electrocardiogram (Yes/No) >70% of patients with suspected HF referred with determination of natriuretic peptides (Yes/No) Use of referral protocols updated in the last 2 years for primary care, emergency room and other specialties (Yes/No) >70% of patients with suspected HF referred with transthoracic echocardiography request (Yes/No) Number of community HF training programs carried out in the last 2 years Number of advanced HF training programs completed in the last 2 years Compliance with the use of referral protocols for primary care (out-of-hospital referrals), in more than 70% of patients (Yes/No)
**Diagnostic**
Existence of non-face-to-face communication systems (telematic consultation, except by telephone) between primary care and specialist (Yes/No) Use of educational support materials about the pathology after diagnosis adapted to patients with HF/caregivers, in more than 70% of cases (Yes/No)
**Treatment**
Existence of an HF unit formed by specialists in cardiology and internal medicine (as well as other specialties if applicable) (Yes/No) Existence and use of treatment adherence programs (Yes/No) % of patients receiving quadruple therapy % of patients achieving drug titration at 6 months % of patients requiring access to cardiac rehabilitation programs that have accessibility Existence of a specific circuit for hospitalized patients diagnosed with HF (regardless of specialty) (Yes/No)
**Follow-up**
Existence of specialized HF nurses (Yes/No) Existence of a specific nursing consultation in the HF unit (with trained staff, exclusive dedication and designated space) (Yes/No) Existence of a nursing figure responsible for centralizing the transitions of patients with HF throughout the care process (case manager) (Yes/No) Use of an integrated care process updated in the last 2 years for patients with HF (Yes/No) Existence of on-demand access for HF patients to the day hospital (Yes/No) Availability of tools for remote follow-up of patients (except by telephone) (Yes/No) Compliance with the use of action protocols for nursing staff in the management of patients with HF greater than 70% (Yes/No) % of patients with HF readmitted in 30 days after hospital discharge less than 11% (Yes/No) Existence of a clear reference for each patient diagnosed with HF, throughout the care process (Yes/No) More than 30% of the consultations are carried out by non-face-to-face follow-up (except by telephone) (Yes/No) Existence of a specific day hospital for HF (Yes/No)

**Table 3:** Identified indicators of transformation to evaluate how the care model evolves.

**Structure**
Integrated area (Hospital Area and Primary Care) Existence of high-resolution consultation in primary care (Yes/No) Cardiac intensive care unit (Yes/No) Unit accredited by Scientific Societies (Yes/No) Number of doctors dedicated to HF (cardiology and internal medicine) Number of healthcare professionals trained and dedicated to the HF multidisciplinary unit Number of nursing professionals trained and dedicated exclusively to HF Number of nursing professionals trained and dedicated exclusively to HF in primary care Existence of the figure responsible in the hospital for centralizing the transitions of the patient with HF throughout the clinical pathway (e.g., case manager) (Yes/No) Existence of e-consultation with primary care (Yes/No) Number of patients admitted by origin (emergency, scheduled, etc.)
**Process**
% of beds occupied by HF patients in the hospital % of confirmed final diagnoses from primary care referrals % of patients who meet the protocol and the criteria for referral to the hospital for suspected HF from primary care Waiting list (in the last 6 months) to receive the first HF diagnostic confirmation visit with the hospital specialist % of patients who are part of health education programs for the management of HF in the hospital and/or primary care % of patients who meet the protocol and the criteria for referral to the hospital for suspected HF from other specialties % of patients with HF (cardiology and internal medicine) who are evaluated in the multidisciplinary clinical case committee of HF Number of training programs in the management of HF, aimed at health professionals of the hospital, carried out in the last 2 years % of patients included in cardiac rehabilitation programs with respect to the total number of patients with HF in the hospital: % in cardiology/% in internal medicine Monitoring of hospital-approved protocols for referral to other hospitals due to the need of other services (hospitals without required technology) % of patients with suspected HF properly referred (including detailed reason, analysis of natriuretic peptides, echocardiography…) % of patients with HF in treatment and follow-up from the multidisciplinary unit, cardiology unit or internal medicine unit with respect to the total % of patients with HF who receive care from cardiology or internal medicine specialists via face-to-face/telephone/video call
**Health outcomes**
% of patients in the HF unit admitted to the intensive care unit due to decompensation Average stay in the cardiology or internal medicine ward of patients admitted with a diagnosis of HF Mortality rate, of any cause, of hospitalized HF patients % of patients with myocardial infarction during hospitalization or post-discharge at 30 days % of HF patients readmitted 30 and 90 days after hospital discharge % of patients with HF post-hospital discharge who have completed a satisfaction survey on the care received for HF management

## Data Availability

The original contributions presented in this study are included in the article/Supplementary Materials. Further inquiries can be directed to the corresponding author.

## Supplementary Materials

The following supporting information can be downloaded at: https://www.mdpi.com/article/10.3390/jcm14103378/s1, Figures S1–S4: General pathways for patients with HF in coordination model 1, corresponding to suspicion, diagnosis, treatment and follow up, respectively; Figures S5–S8: General pathways for patients with HF in coordination model 2, corresponding to suspicion, diagnosis, treatment and follow up, respectively; Figures S9–S12: General pathways for patients with HF in coordination model 3, corresponding to suspicion, diagnosis, treatment and follow up, respectively; Figures S13–S16: General pathways for patients with HF in coordination model 4, corresponding to suspicion, diagnosis, treatment and follow up, respectively.

## Appendix A

The CARABELA-HF Scientific Committee consists of the following members: Álvaro González Franco (Sociedad Española de Medicina Interna, SEMI), Inmaculada Mediavilla (Sociedad Española de Calidad Asistencial), José Francisco Soto (Sociedad Española de Directivos de la Salud, SEDISA), Juana Carretero (SEMI), Julián Pérez-Villacastín (Sociedad Española de Cardiología, SEC), Manuel Anguita (SEC), Lucía Regadera (AstraZeneca Farmacéutica Spain), Alberto Prado Dominguez (AstraZeneca Farmacéutica Spain), Manuel Leal (AstraZeneca Farmacéutica Spain), and Victoria González Pastor (AstraZeneca Farmacéutica Spain).
