# Supplementary material for: Healthcare Models and Quality Indicators in the Management of Patients with Heart Failure in Spain: Results from the CARABELA-HF Initiative
Source: J Clin Med. 2025 May 12;14(10):3378. doi: 10.3390/jcm14103378 (PMC12112352; doi:10.3390/jcm14103378)

Figure S1. SUSPICION
Coordination model 1: HF unit comprised of Cardiology and Internal Medicine

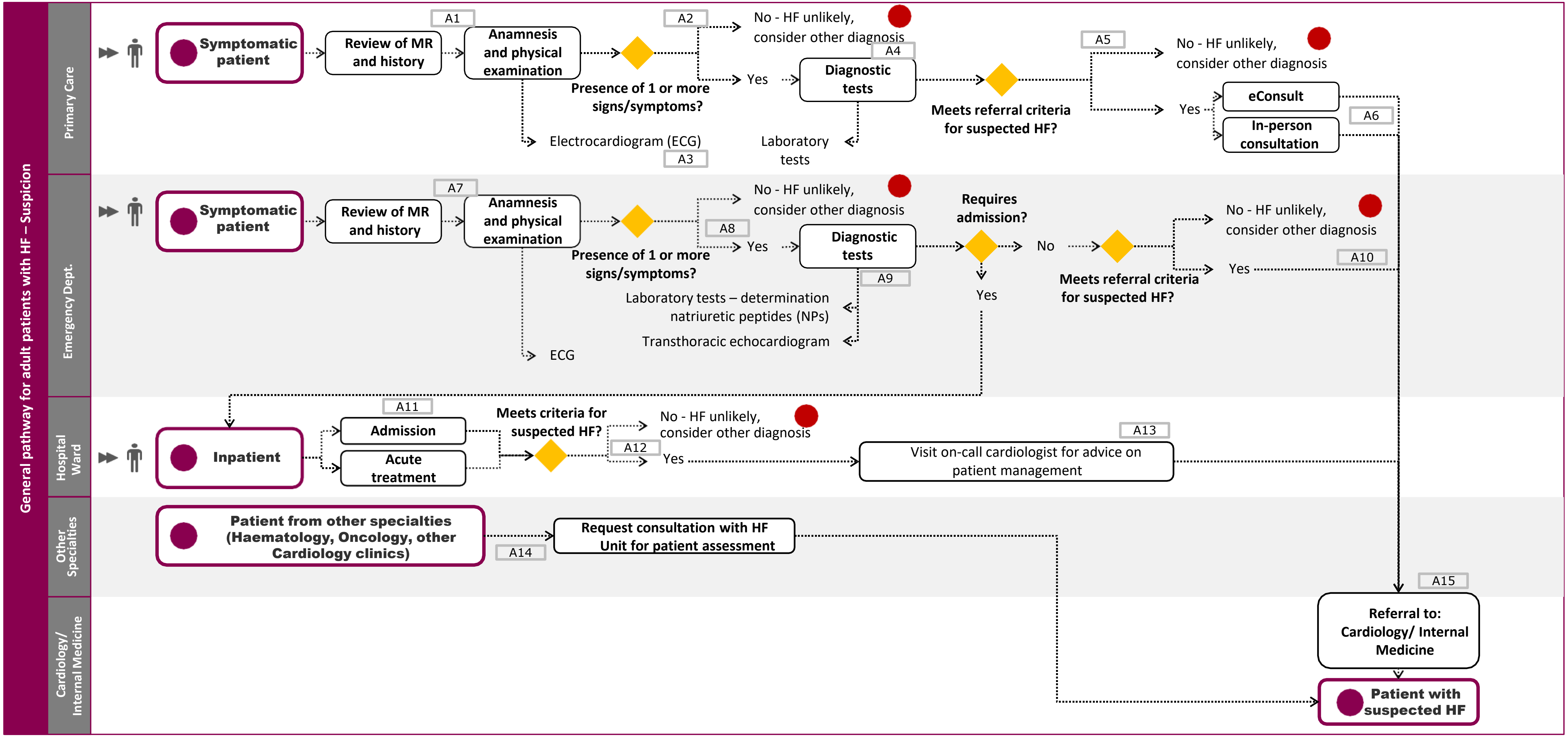

Figure S2.DIAGNOSIS
Coordination model 1: HF unit comprised of Cardiology and Internal Medicine

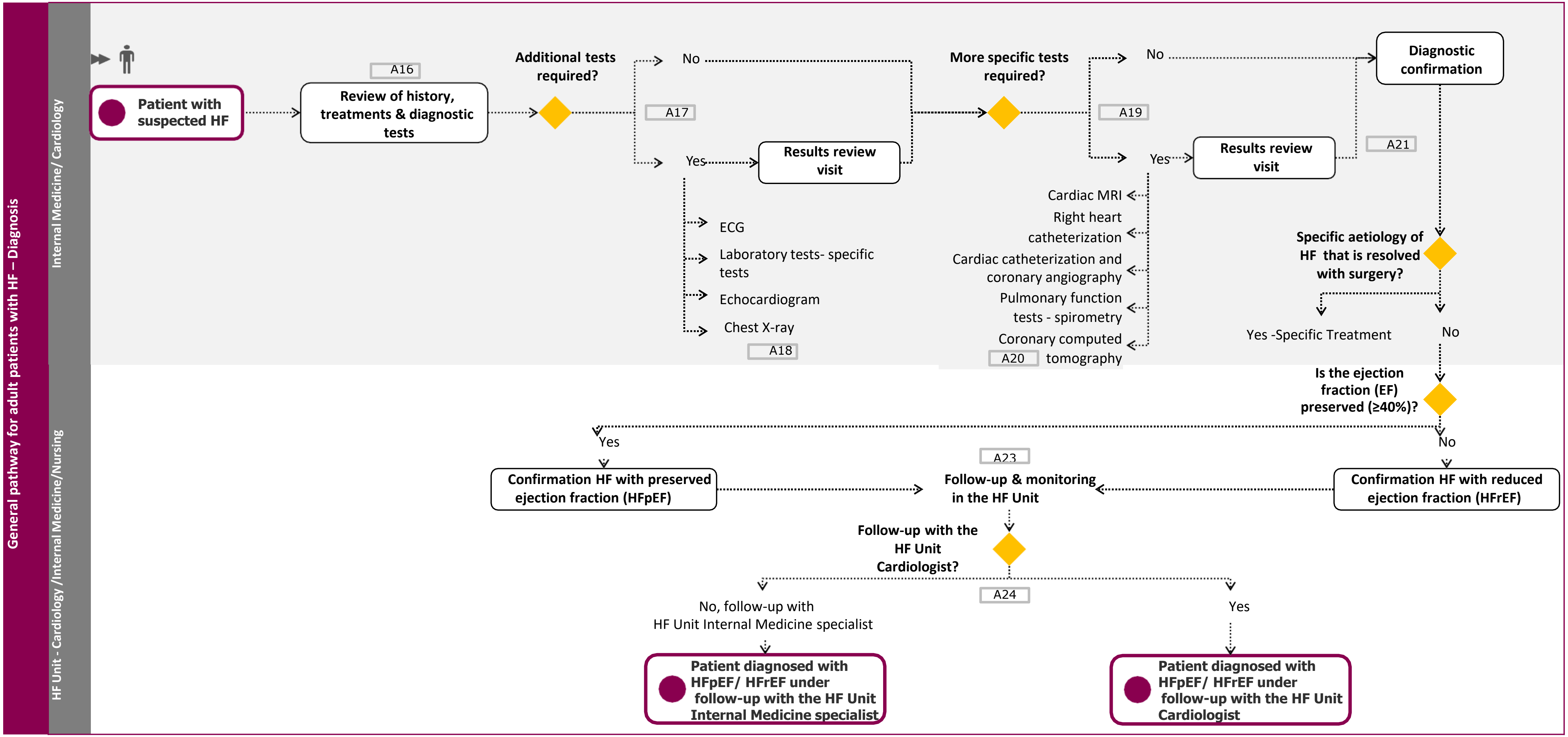

Figure S3. TREATMENT

Coordination model 1: HF unit comprised of Cardiology and Internal Medicine

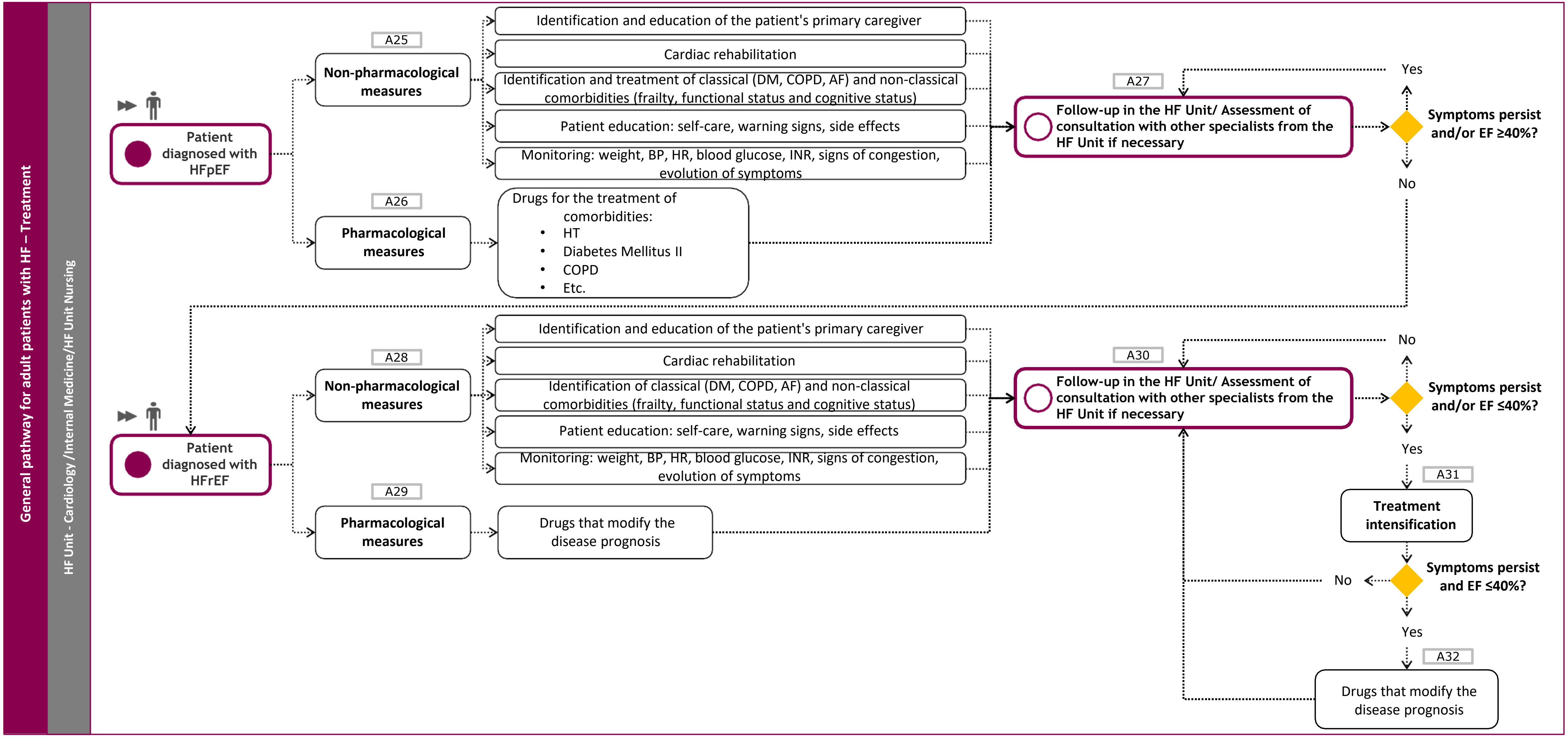

Figure S4.FOLLOW-UP
Coordination model 1: HF Unit comprised of Cardiology and Internal Medicine

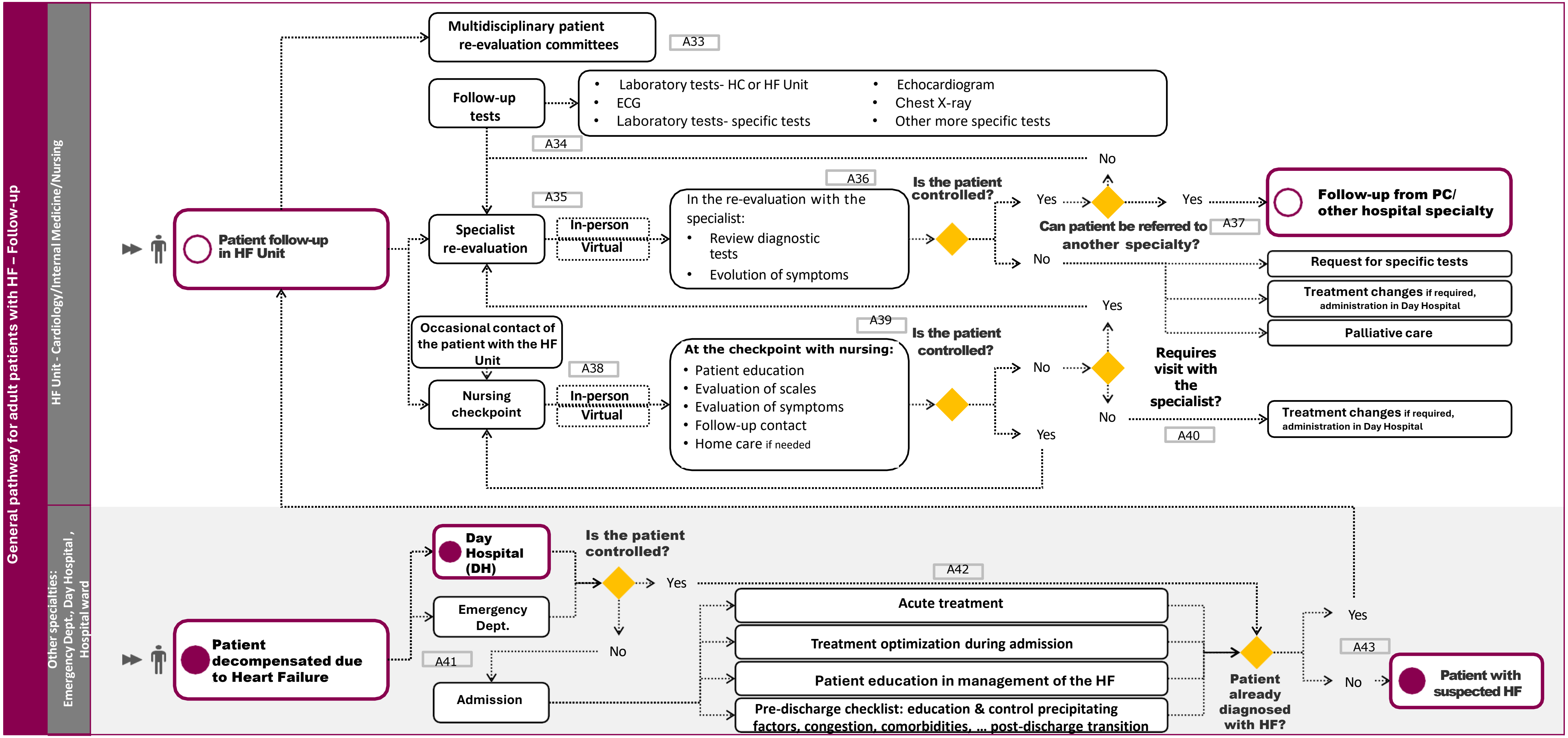

Figure S5. SUSPICION

Coordination model 2: Independent HF Unit in Cardiology and Independent HF Unit in IM

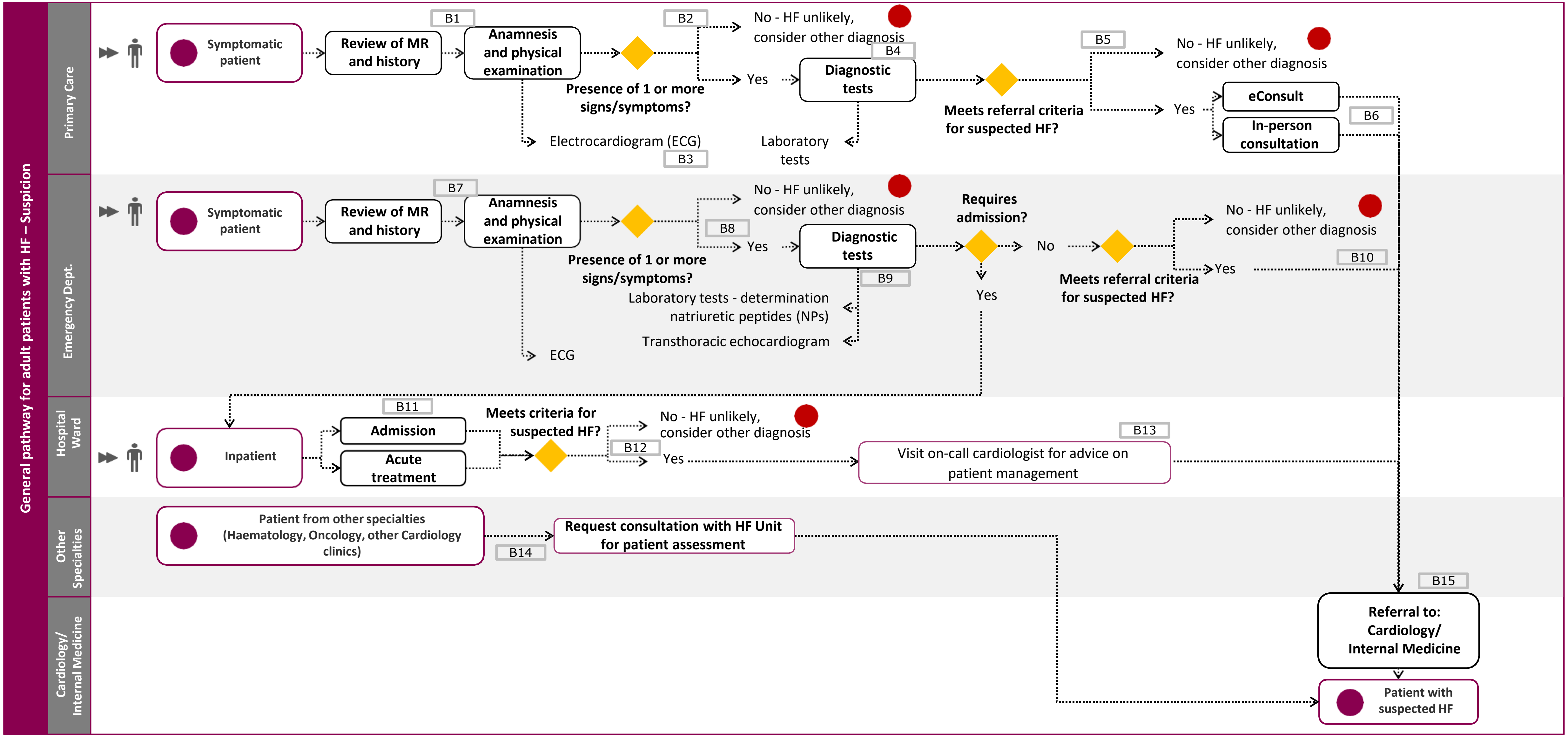

Figure S6. DIAGNOSIS
Coordination model 2: Independent HF Unit in Cardiology and independent HF Unit in IM

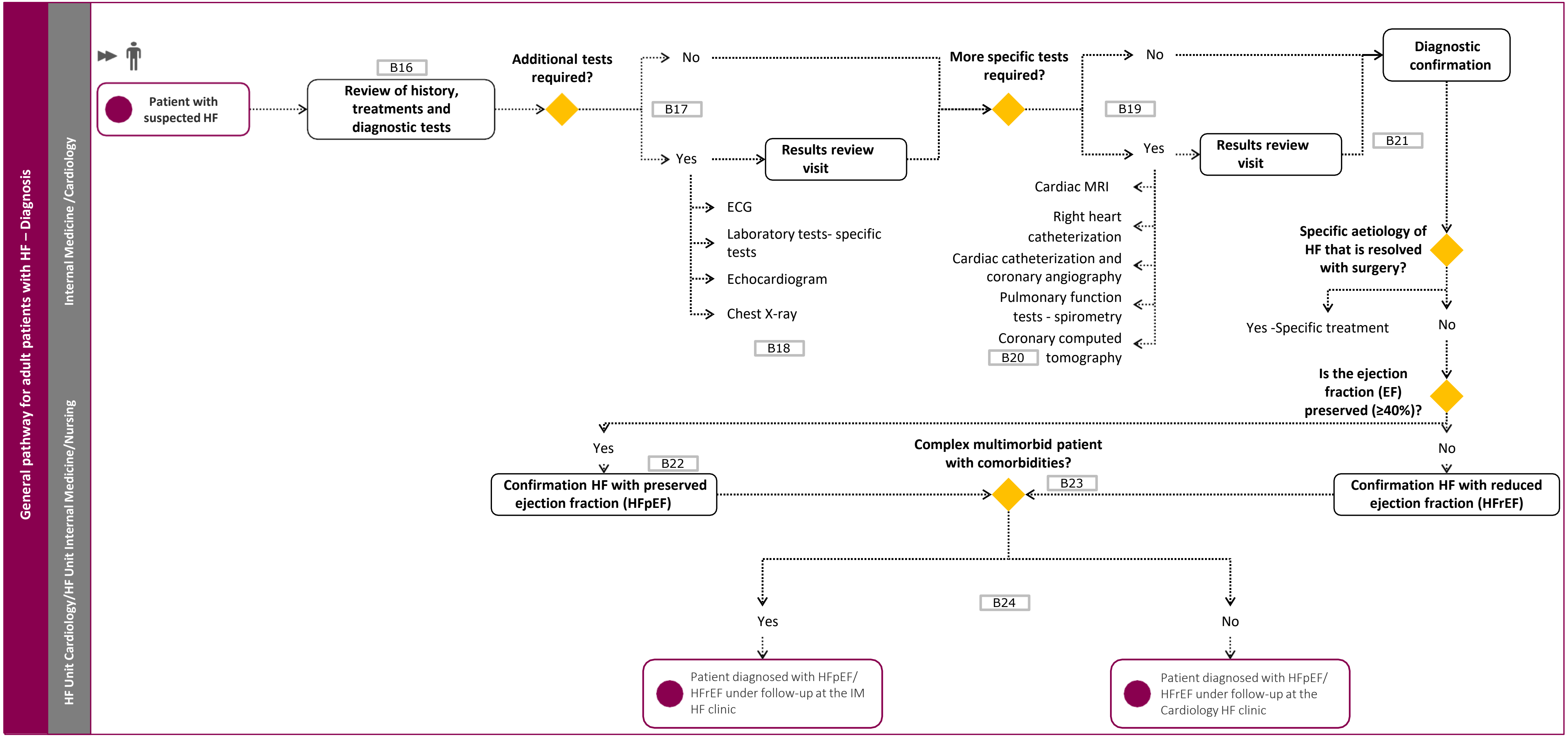

Figure S7. TREATMENT
Coordination model 2: Independent HF Unit in Cardiology and Independent HF Unit in IM

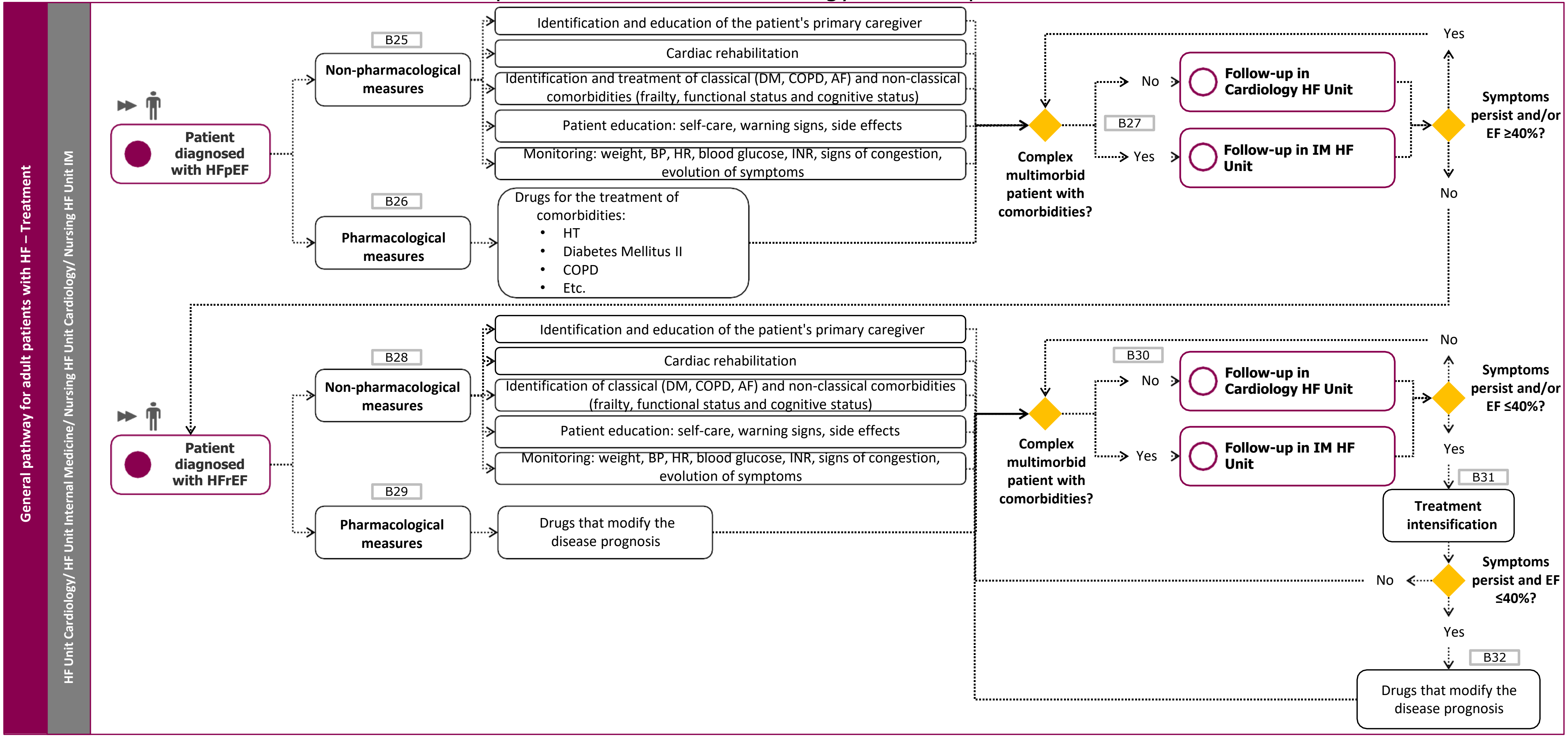

Figure S8. FOLLOW-UP
Coordination model 2: Independent HF Unit in Cardiology and Independent HF Unit in IM

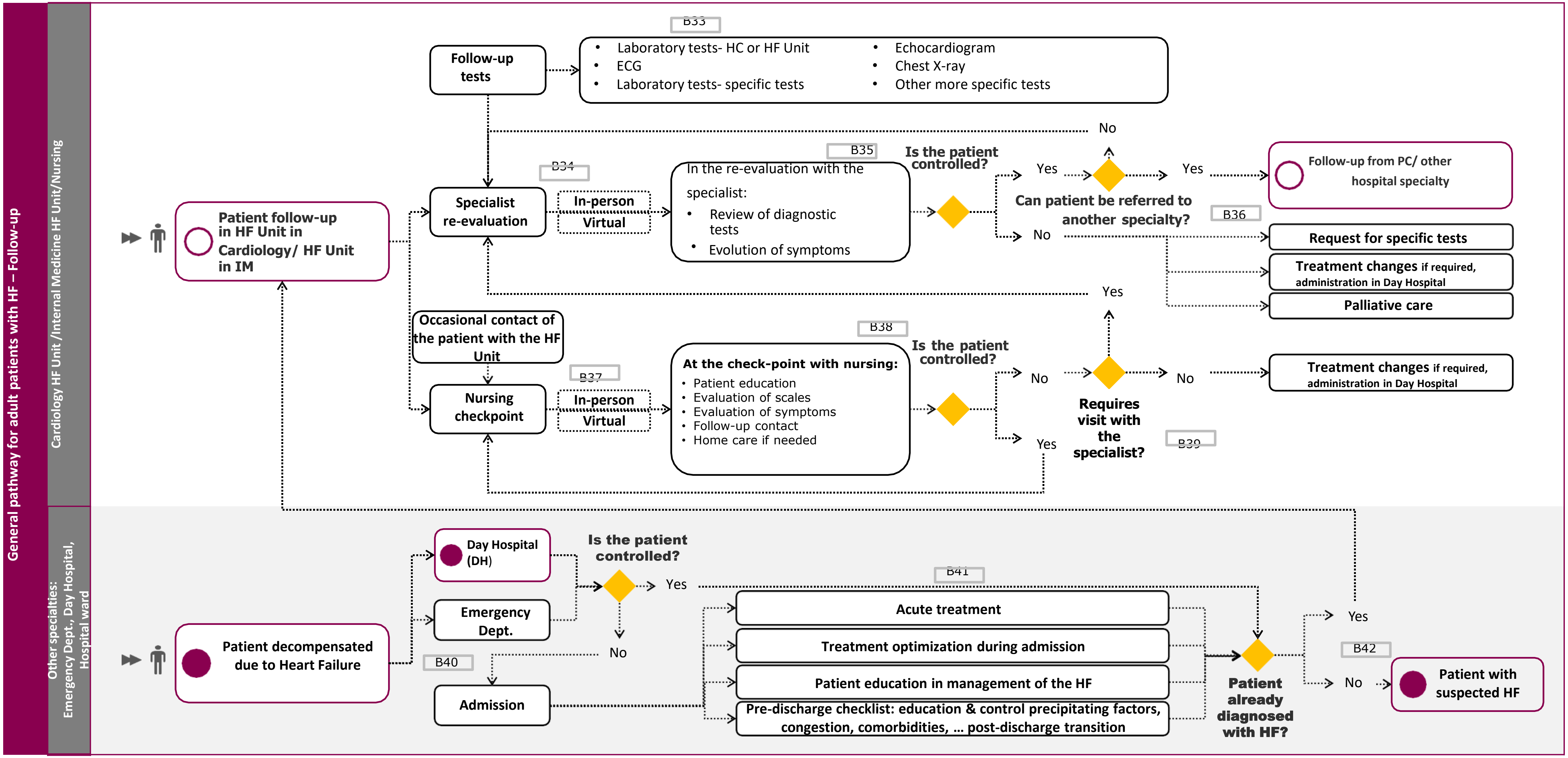

Figure S9. SUSPICION
Coordination model 3: HF Unit in Cardiology and independent HF clinic in IM

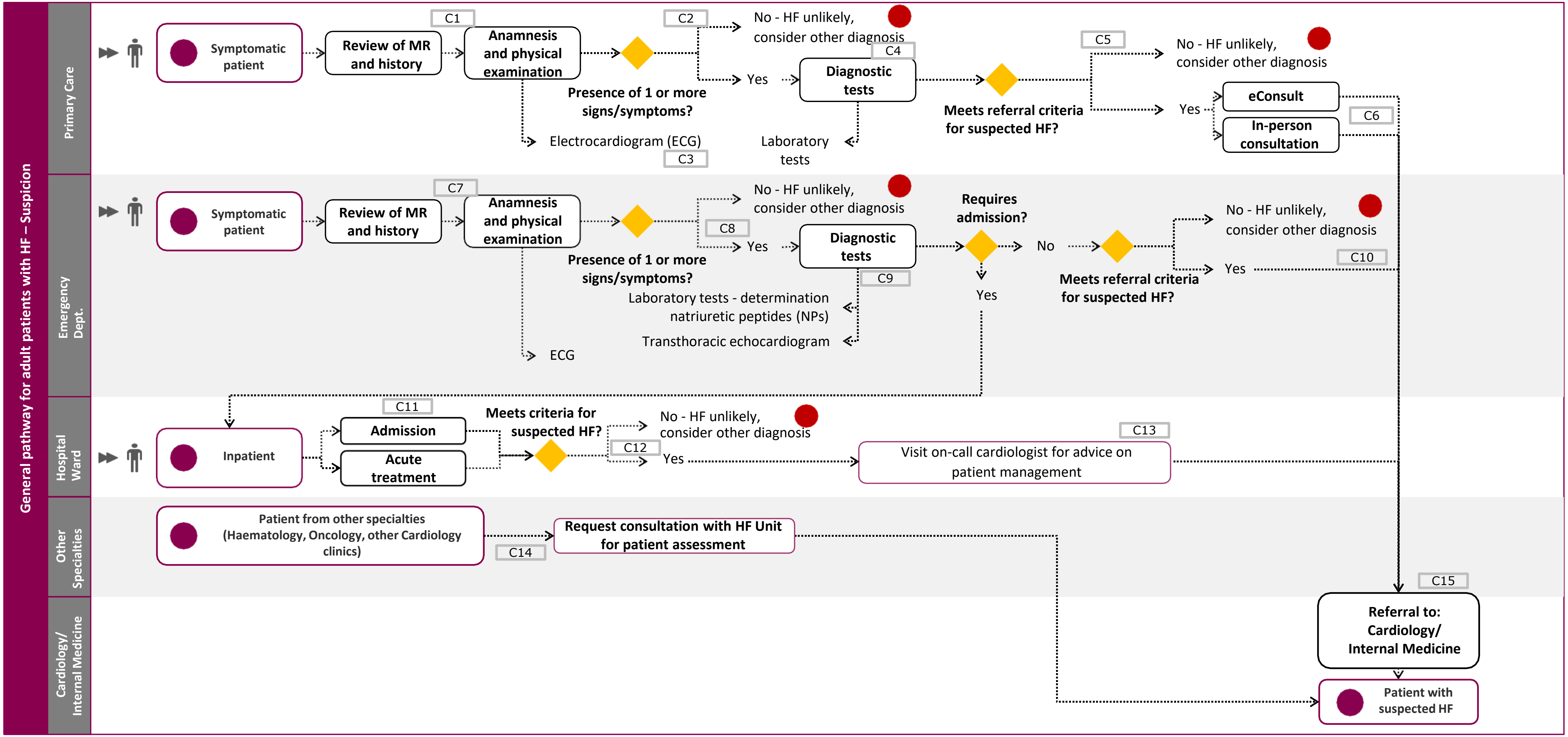

Figure S10. DIAGNOSIS
Coordination model 3: HF Unit in Cardiology and independent HF clinic in IM

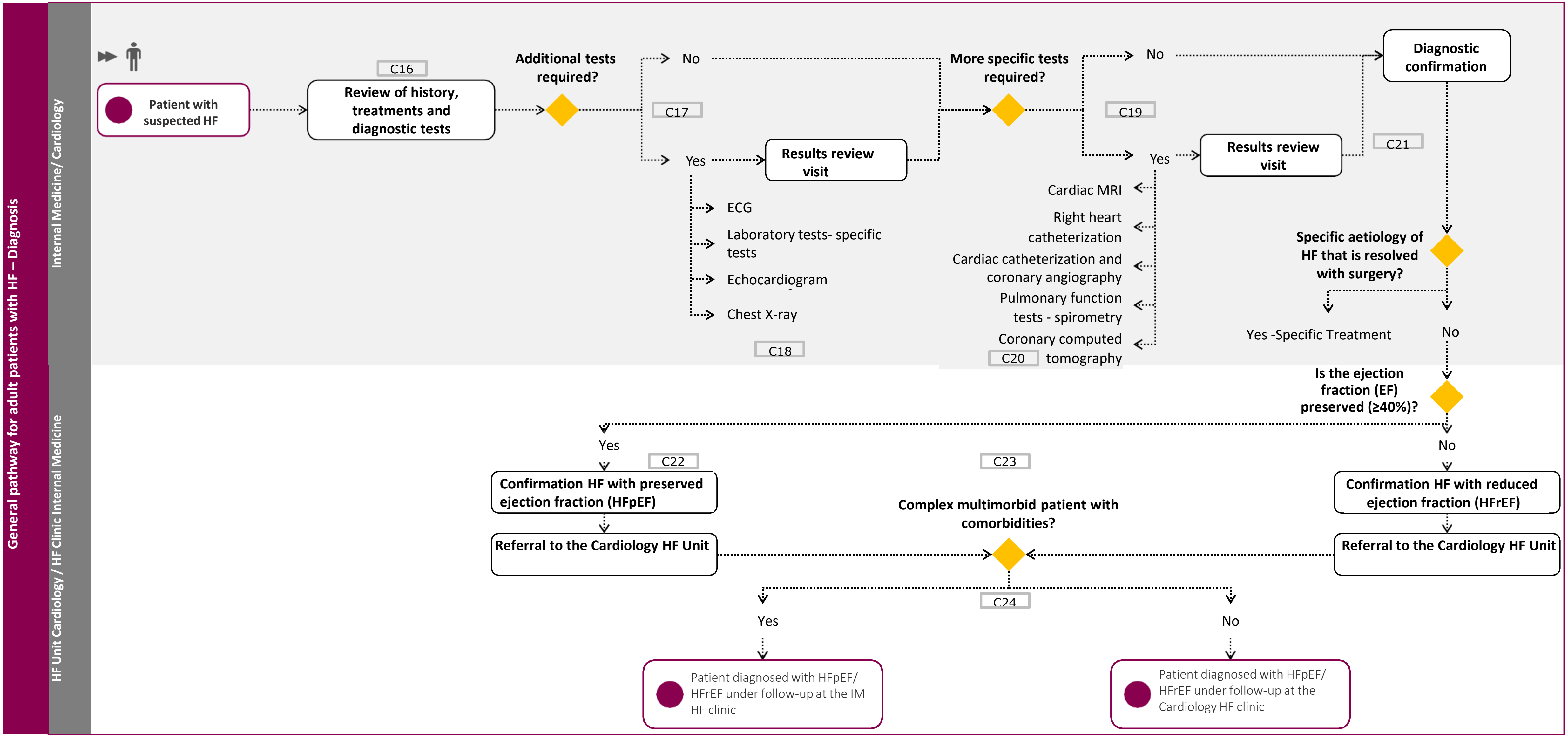

Figure S11. TREATMENT

Coordination model 3: HF Unit in Cardiology and independent HF clinic in IM

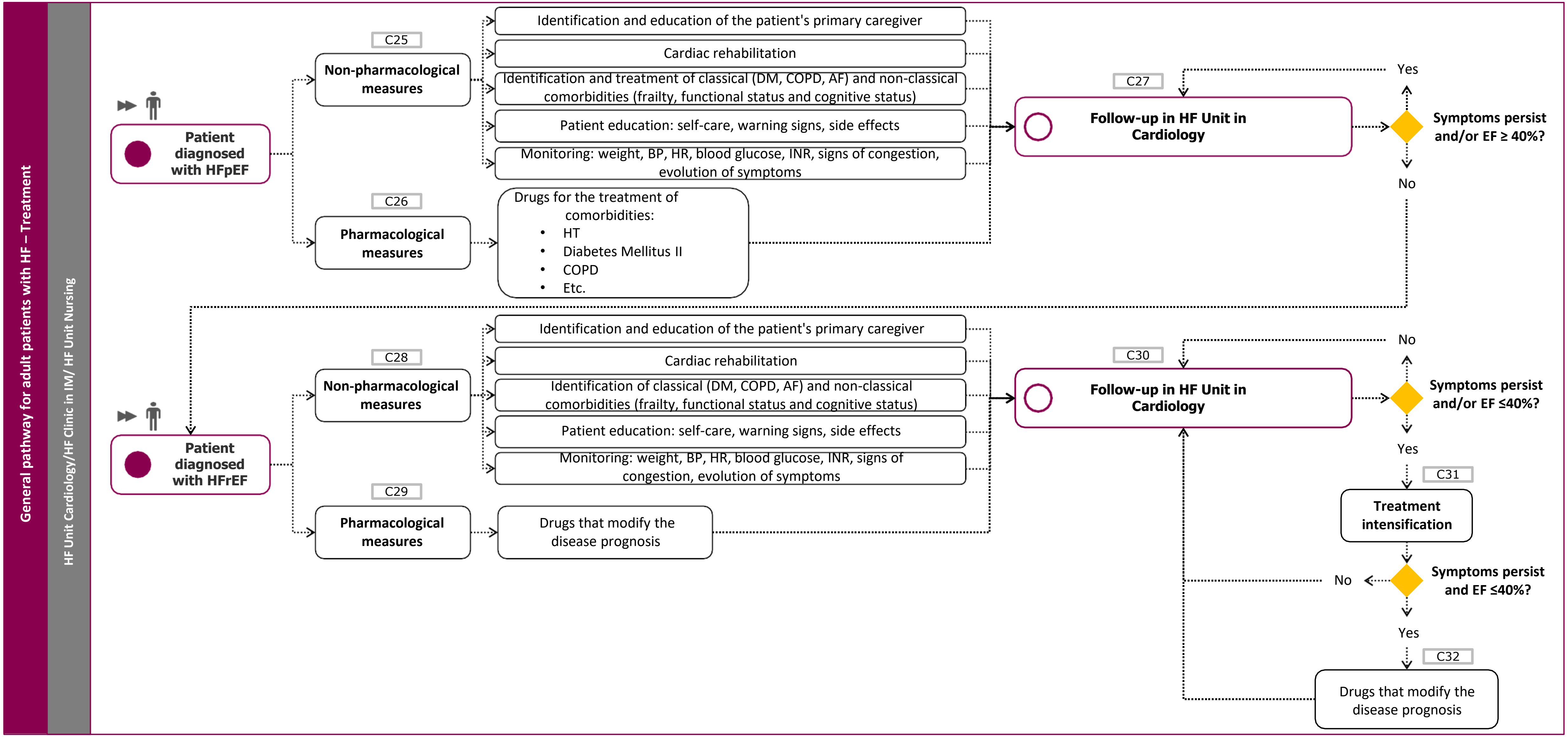

Figure S12. FOLLOW-UP
Coordination model 3: HF Unit in Cardiology and independent HF clinic in IM

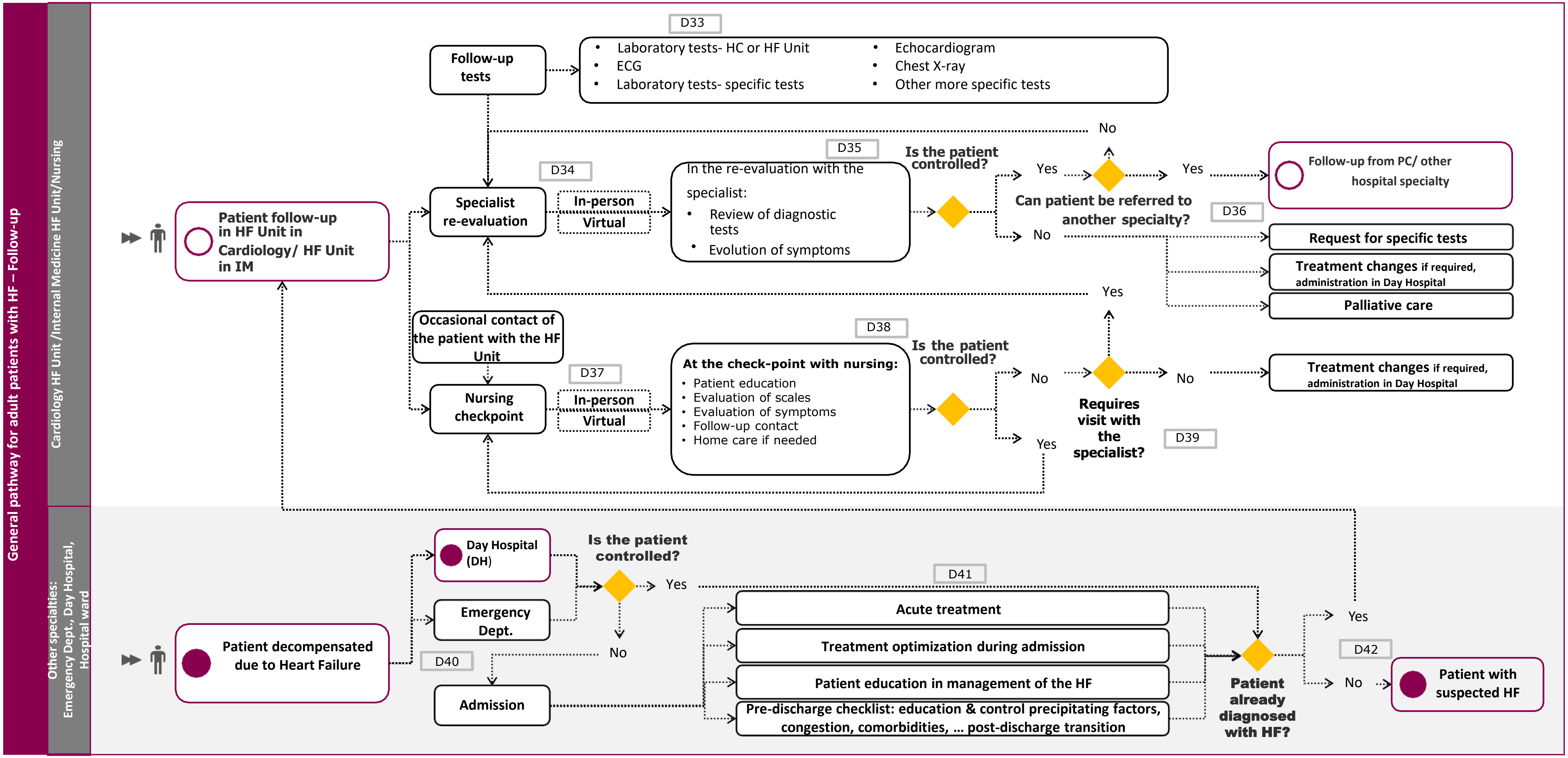

Figure S13. SUSPICION
Coordination model 4: Cardiology HF Clinic and IM HF Clinic

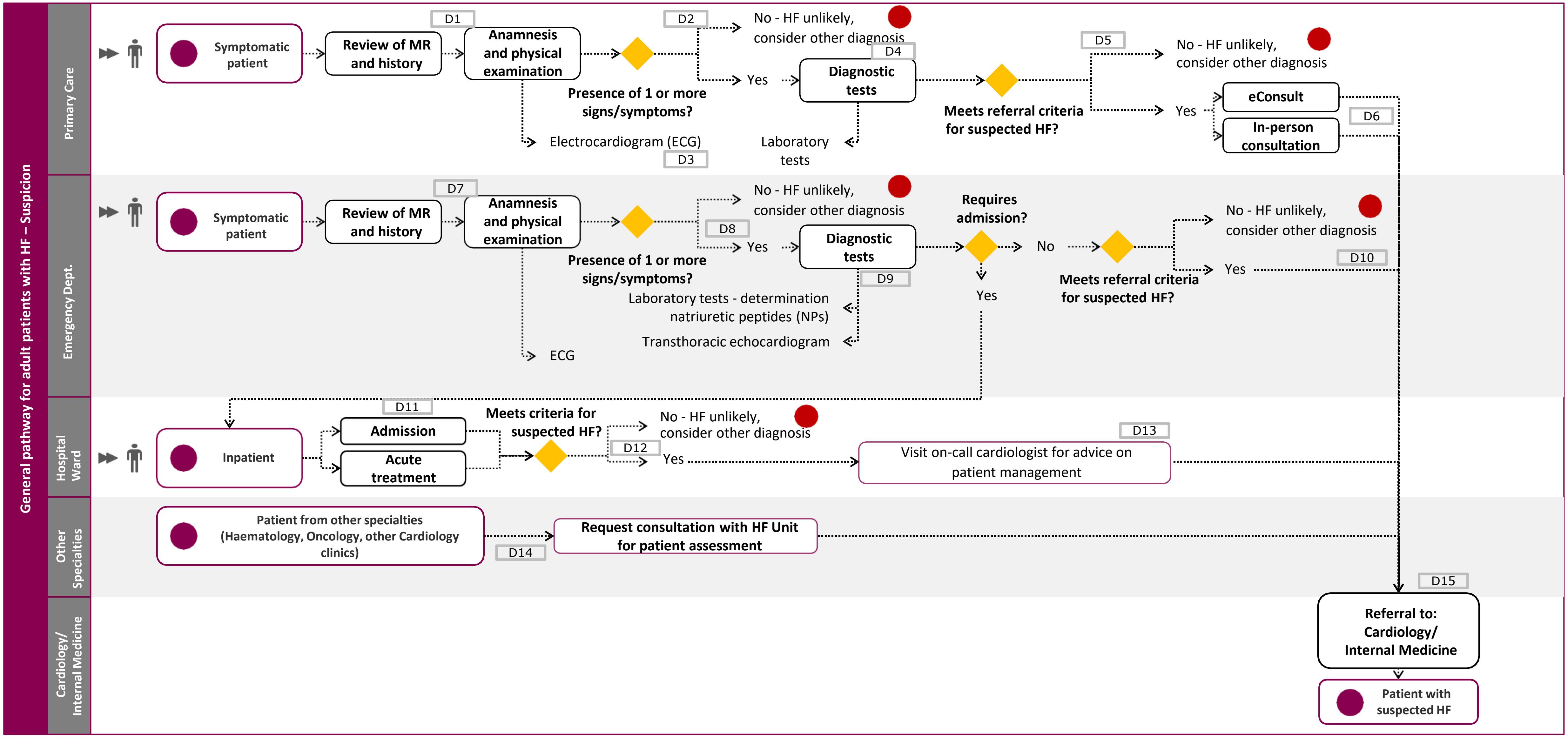

Figure S14. DIAGNOSIS
Coordination model 4: Cardiology HF Clinic and IM HF Clinic

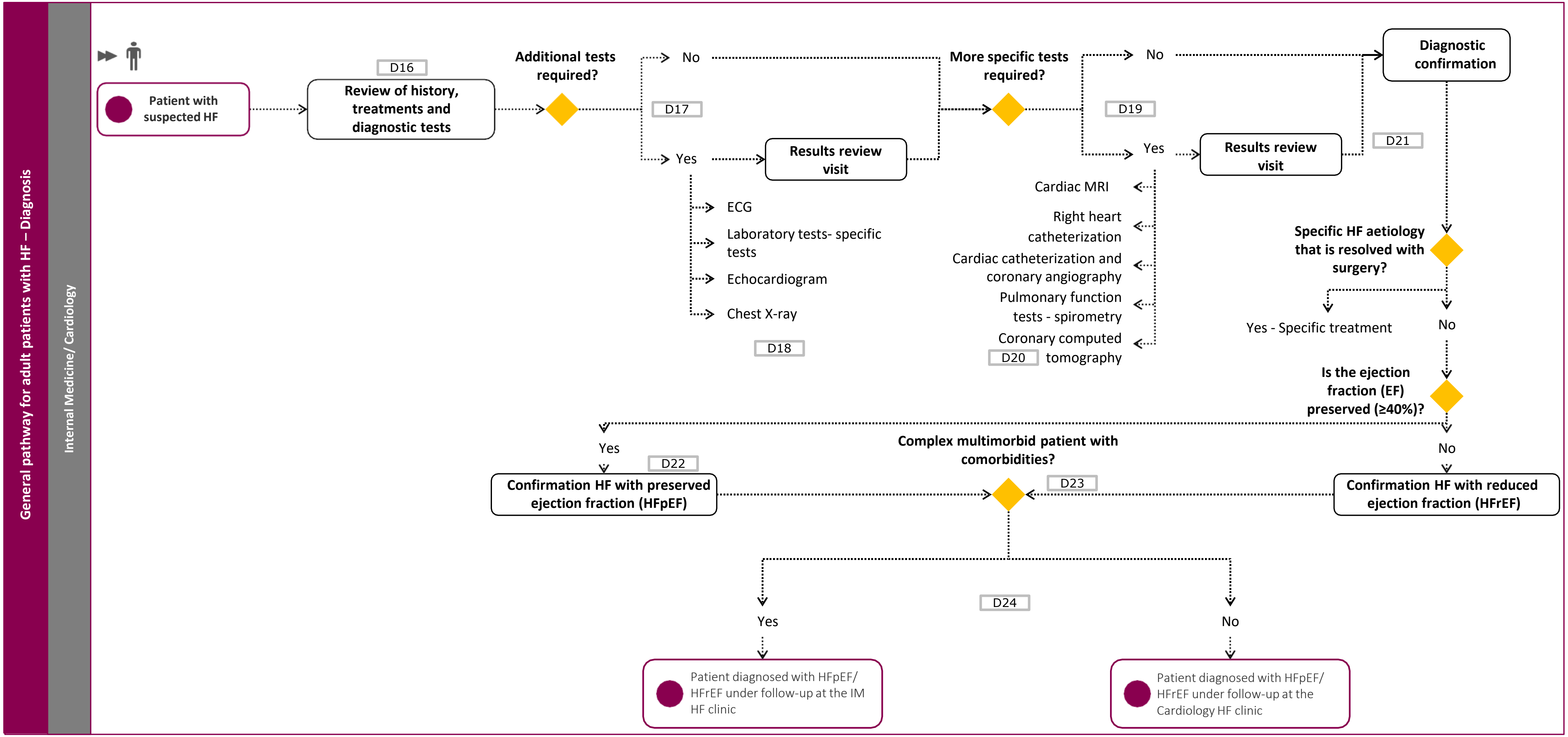

Figure S15. TREATMENT
Coordination model 4: Cardiology HF Clinic and IM HF Clinic

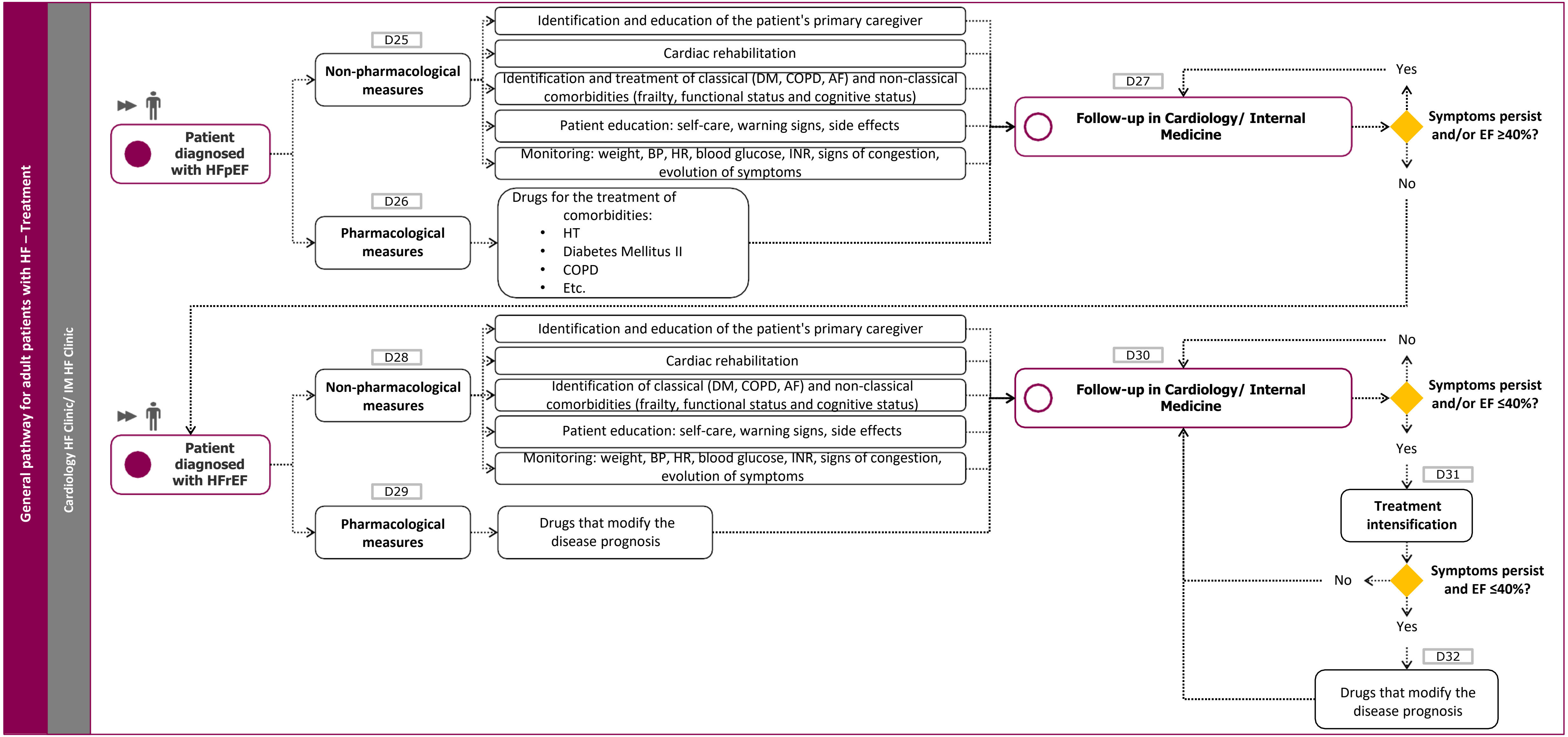

Figure S16. FOLLOW-UP
Coordination model 4: Cardiology HF clinic and IM HF clinic

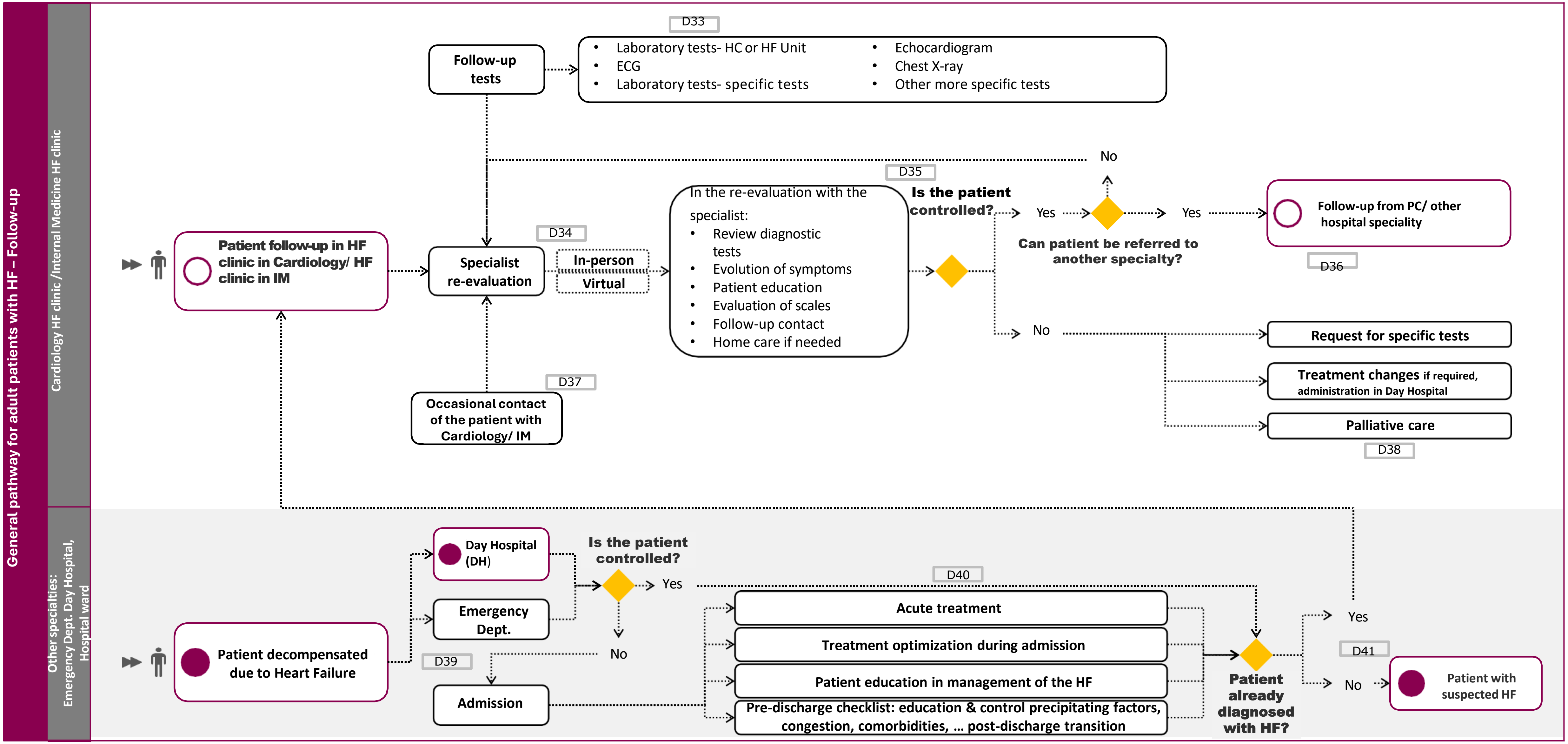

Supplement: Supplementary file 1 [file jcm-14-03378-s001.zip › jcm-3568154-supplementary.pdf]
